# Evolutionary responses to climate change in a range expanding plant

**DOI:** 10.1007/s00442-017-3864-x

**Published:** 2017-04-13

**Authors:** Mirka Macel, Tomáš Dostálek, Sonja Esch, Anna Bucharová, Nicole M. van Dam, Katja Tielbörger, Koen J. F. Verhoeven, Zuzana Münzbergová

**Affiliations:** 1 Plant Ecology, University of Tübingen, Auf der Morgenstelle 5, 72076 Tübingen, Germany; 2 Department of Botany, Faculty of Science, Charles University in Prague, Benátská 2, 128 01 Prague 2, Czech Republic; 3 Institute of Botany, The Czech Academy of Sciences, Zámek 1, 252 43 Průhonice, Czech Republic; 4 Institute of Biology, Leiden University, P.O. Box 9505, 2300 RA, Leiden, The Netherlands; 5 Plant Evolutionary Ecology, University of Tübingen, Auf der Morgenstelle 5, 72076 Tübingen, Germany; 6 Molecular Interactions Ecology, German Centre for Integrative Biodiverisity Research (iDiv), Deutscher Platz 5e, 04103 Leipzig, Germany; 7 Institute of Ecology, Friedrich Schiller University Jena, Dornburger-Str. 159, 07743 Jena, Germany; 8 Molecular Interaction Ecology, Department of Plant Science, Radboud University, P.O. Box 9010, 6500 GL Nijmegen, The Netherlands; 9 Terrestrial Ecology, Netherlands Institute of Ecology (NIOO-KNAW), Droevendaalsesteeg 10, 6708 PB Wageningen, The Netherlands

**Keywords:** Global change, Herbivores, Biotic interactions, Insects, *Rorippa austriaca*

## Abstract

To understand the biological effects of climate change, it is essential to take into account species’ evolutionary responses to their changing environments. Ongoing climate change is resulting in species shifting their geographical distribution ranges poleward. We tested whether a successful range expanding plant has rapidly adapted to the regional conditions in its novel range, and whether adaptation could be driven by herbivores. Furthermore, we investigated if enemy release occurred in the newly colonized areas and whether plant origins differed in herbivore resistance. Plants were cloned and reciprocally transplanted between three experimental sites across the range. Effects of herbivores on plant performance were tested by individually caging plants with either open or closed cages. There was no indication of (regional) adaptation to abiotic conditions. Plants originating from the novel range were always larger than plants from the core distribution at all experimental sites, with or without herbivory. Herbivore damage was highest and not lowest at the experimental sites in the novel range, suggesting no release from enemy impact. Genotypes from the core were more damaged compared to genotypes from newly colonized areas at the most northern site in the novel range, which was dominated by generalist slug herbivory. We also detected subtle shifts in chemical defenses between the plant origins. Genotypes from the novel range had more inducible defenses. Our results suggest that plants that are expanding their range with climate change may evolve increased vigor and altered herbivore resistance in their new range, analogous to invasive plants.

**Electronic supplementary material:**

The online version of this article (doi:10.1007/s00442-017-3864-x) contains supplementary material, which is available to authorized users.

## Introduction

Species distributions are currently changing through globalization and climate change (Parmesan and Yohe 2003; Gonzalez-Megias et al. 2008; Chen et al. 2011). For example, global warming is shifting species distribution limits towards the poles (Hickling et al. 2006). Not all species’ distributions will change at the same speed, if they can change at all (Berg et al. 2010). Therefore, current range expansions or range shifts may lead to shifts in biotic interactions because interacting organisms may not move all at the same pace (Van der Putten et al. 2010). Range expanding species are thus likely to encounter different abiotic as well as biotic environments in their new range. These novel selection pressures during range expansion could lead to rapid adaptive evolution of the species in the new range (Buckley and Bridle 2014). Understanding how species respond to these new abiotic and biotic environments is crucial to our knowledge about the biological effects of global warming.

Evolutionary processes can differ between the core and edge populations of a species’ range (Kirkpatrick and Barton 1997). As an example, evolution in range edge populations is thought to be affected by the level of gene flow from core populations, where adaptation in the range edge can be constrained by too little gene flow (providing insufficient variation for adaptive responses to selection) or too much gene flow (swamping incipient adaptation). For currently range expanding species, both evolutionary and ecological processes in the populations in the new extended range may, therefore, be different compared to the native range (Sexton et al. 2009). Shifts in selection on species traits by the new abiotic and biotic environments may be strong enough to lead to adaptive differentiation between the new range edge and the native range. For example, plant–herbivore interactions can change, causing novel and strong selection on plant traits such as defenses (Nylund et al. 2012). Loss or partial loss of natural enemies can occur during range expansion, which can further promote successful range expansion (Menendez et al. 2008; Lakeman-Fraser and Ewers 2013). In addition, some insects are moving to novel host plants in their extended range and have adapted to these new host species (Buckley and Bridle 2014). Multiple examples from invasion biology illustrate that altered plant–herbivore interactions can cause a shift in chemical defenses as well as plant growth (e.g., Blossey and Nötzold 1995; Joshi and Vrieling 2005). For instance, recent studies showed that rapid evolution of invasive plant populations led to higher resistance to belowground herbivores and higher toxin levels (Zheng et al. 2015) or better defense against aboveground generalist herbivores but greater susceptibility to specialists compared to native genotypes (Lin et al. 2016).

Thus far, little is known about (adaptive) evolution of plants that are currently expanding their range due to global warming. Adaptation of plants to their local environments can be tested using reciprocal transplant approaches. Higher performance of genotypes in their home environment compared to foreign environments and/or higher performance of the local genotypes compared to the foreign genotypes within a site would indicate local adaptation (e.g., Joshi et al. 2001; Kawecki and Ebert 2004). Using such comparisons, the contribution of environmental and genetic factors to species performance can be assessed. Such approaches can also be used to test the performance of plants outside of their present range by including an experimental transplant site that is located in the future predicted range (Marsico and Hellmann 2009; Lakeman-Fraser and Ewers 2013). Although there have been many transplant studies on plant local adaptation (reviewed in Leimu and Fischer 2008), not many of these field studies have experimentally assessed specific biotic or abiotic factors driving local adaptation in plants (but see Macel et al. 2007; Bischoff and Hurault 2013; Pankova et al. 2014). Here, we used a reciprocal transplant approach with herbivore manipulation to test whether a range expanding plant species has rapidly adapted to the environment in its novel range, and whether such adaptation is driven mainly by interactions with herbivores or by other factors such as abiotic conditions.

Our study species is Austrian yellow-cress, *Rorippa austriaca* (Crantz) Besser (Brassicaceae). Like many species that have expanded their range poleward due to climate change (Chen et al. 2011), this perennial plant has expanded its range northward from its original distribution in southeastern Europe to the current northern range limit in southern Scandinavia (Bleeker 2003). *Rorippa austriaca* was first recorded in the Netherlands between 1900 and 1925 and has increased in abundance there in the last decades (Tamis et al. 2004). In addition to climate change, the increasing connection of waterways in Europe may have contributed to its range expansion. The spread of the species occurs mostly naturally along rivers. Human-aided spread can happen through redistributions of soils (Keil 1999). Habitats where *R. austriaca* occurs in its novel range are riversides, ruderal areas and agricultural fields (Bleeker 2003, Table S1). It has been considered invasive in parts of its new range, e.g., Germany and the Netherlands, where it also hybridizes with native *Rorippa* species (Bleeker 2003; Engelkes et al. 2012). *Rorippa austriaca*, like most Brassicaceae, contains glucosinolates as defense compounds (Huberty et al. 2014). The species has also been introduced from Europe to North America. Plants of invasive populations in North American grew bigger than native Austrian plants but showed no difference in resistance to generalist slug herbivory (Buschmann et al. 2005). We used plants from three European regions, two regions that are recently colonized, the Netherlands (five populations) and southern Germany (two populations) and one region at the core distribution, the Czech Republic (five populations), and had an experimental site in each of the regions. The plants from the Czech Republic were from the western edge of the core distribution. We planted the same ten individuals of each population, propagated via root cuttings, in each site. To test the effect of insect communities and gastropods on the plants in the experimental sites, all plants were caged individually with open or closed cages to either include or exclude insects and gastropods. Plant performance, herbivore damage/presence, and chemical defenses were measured for one growing season. Our aim was to investigate (a) whether plants would perform better in their home region (regional adaptation) with and without herbivory, (b) whether herbivory was less in the novel range compared to the core edge region due to potential enemy release in the recently colonized range, and (c) whether plant origins differed in herbivory and chemical defenses.

## Materials and methods

### Reciprocal transplant experiment

**Fig. 1:**
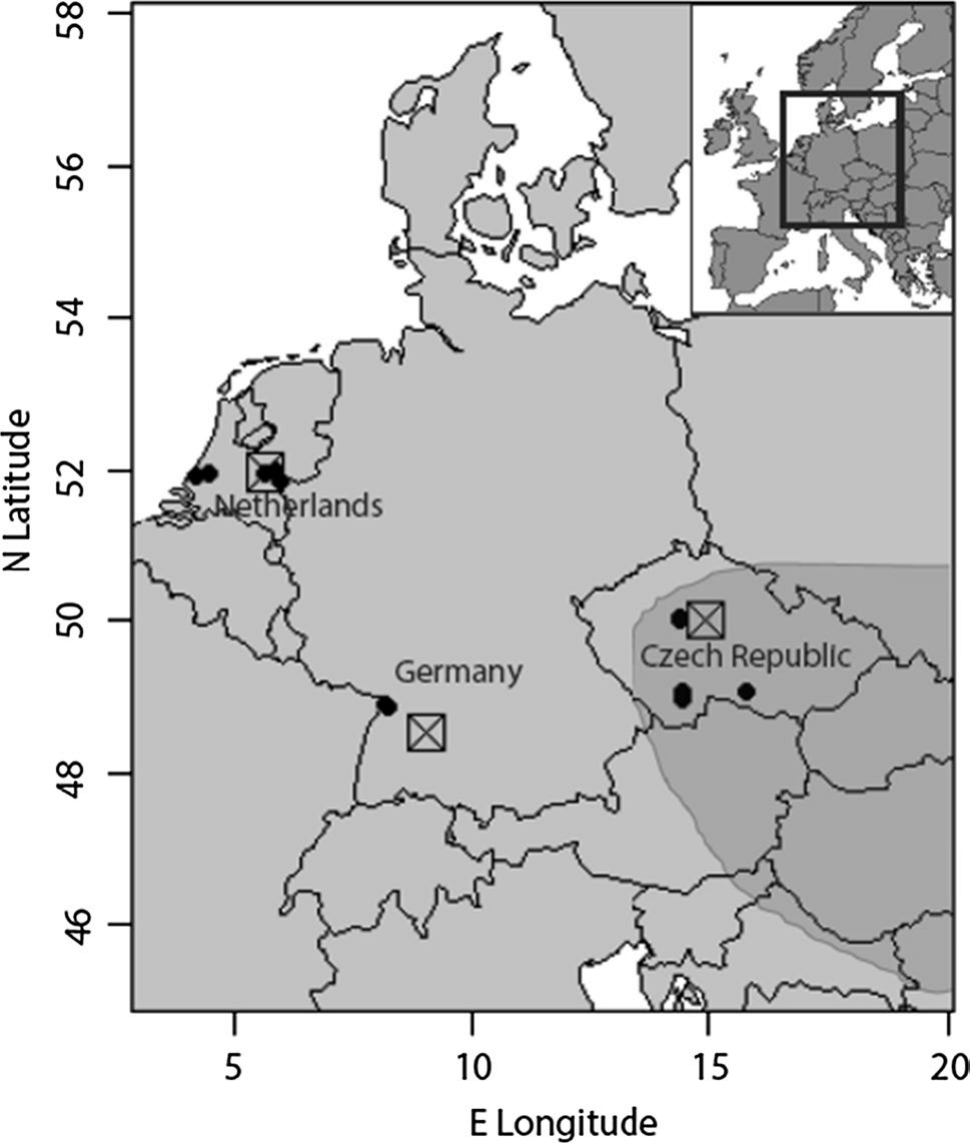
Map of the *Rorippa austriaca* populations of the Czech Republic, Germany and the Netherlands used for the experiment (*small solid black dots*) and experimental sites in each region (*open squares*). *The darker gray fill* roughly depicts the original core distribution of the species, although its precise borders are unknown (modified after Bleeker 2003). Populations in the Czech Republic are at the edge of the core distribution. Germany, the Netherlands, Scandinavia and other parts of Western Europe are newly colonized areas. For exact coordinates of the *R. austriaca* populations, see Table S1

Plants of *R. austriaca* from seven populations of the novel range and five populations of the core edge were collected in the field between May and October 2011 by digging out parts of the roots. In each population, a minimum of 10 plants were collected which grew at least 5 m apart. Five populations were sampled in the Netherlands (novel range), two populations in southern Germany (novel range), and five populations in the Czech Republic (core edge) (see Fig. 1, Table S1 and Huberty et al. 2014; Dostalek et al. 2016). For comparisons between core populations and populations of the novel range, ideally the source populations from which the novel populations originated should be compared. Population genetic analyses using SNPs showed that the core edge Czech populations were more related to the German and Dutch populations in the novel range than *R. austriaca* populations from the core distribution in Hungary or Romania (Macel M. et al., unpublished data). All plants were checked for ploidy levels with flow cytometry, and were confirmed to be diploid with genome size corresponding to *R. austriaca*. Potential hybrids between *R. austriaca* and other *Rorippa* species would have been polyploid (Bleeker and Matthies 2005). All field-collected plants were first grown in the same soil, 1:1 mixture of potting soil and sand, in 3 L pots in a common garden in Tübingen (Germany) for 7 months. Each plant was then propagated via root cuttings at the end of April 2012. Six root pieces of 3 mm diameter × 5 cm length were cut off per plant. Two root cuttings of each plant were sent by mail to the Czech Republic and two cuttings per plant to the Netherlands.

Three experimental common gardens were set up in May 2012: Wageningen (The Netherlands) 51°58′N 5°40′E, Tübingen (Germany) 48°31′N 9°03′E, and Průhonice (Czech Republic) 50°0′N 14°56′E (Fig. 1). Average temperatures in the regions during the course of the experiment were 16.3° C (Wageningen), 17.9° C (Tübingen) and 17.6° C (Průhonice). Average monthly precipitation was 87 mm (Wageningen), 66 mm (Tübingen) and 74 mm (Průhonice) (sources: www.knmi.nl, www.wetterkontor.de, www.chmi.cz). These experimental sites were in each of the three regions of plant origin, and distances between the regional populations used in the experiment and the experimental sites were: Wageningen 4–99 km, Tübingen 71–75 km, Průhonice 11–139 km. In each experimental site, two clones (two root cuttings) of each plant were grown, in total 240 plants per site (12 populations × 10 plants × 2 clones). Each root cutting was planted in a 3 L pot with 1:1 sand—potting soil mixture. Pots were placed 75 cm apart in a 12 × 15 m fenced-off plot in a completely randomized design. The plot was covered with root foil (polypropylene) to suppress the growth of other plants in the plot. The pots were watered when needed in the beginning of the experiment (first month) to enable good establishment of the plants. All plants were covered with mesh bags (polyamide, 0.75 m width × 1.20 m length, 0.7 mm × 0.8 mm mesh size), but for half of the plants (one of the two clones) the mesh bags were opened on one vertical side. The mesh bags on the other half of the plants were kept closed to exclude most insects and other herbivores. The mesh bags were held upright by bamboo and iron sticks.

During the growing season, plant performance was measured twice, once in middle of the season (end of June) and once at the end of August. Herbivore damage, plant size and number of flowers were recorded. Height of the longest shoot was measured and the number of side shoots and flowering shoots counted. Percentage total herbivore damage was estimated on a standardized subset of seven leaves of each plant; the youngest and oldest fully grown leaves and five randomly chosen in between, and the average of these seven leaves was used in the analyses. Additionally, at the Czech site the percentage leaf area infected with spider mites (leaf area covered with spider mite webs) was estimated on the same seven leaves. The other type of leaf damage was roughly estimated as fraction of total damage (spider mite infection excluded) in three types: (1) shot holes caused by adult flea beetles (Chrysomelidae), (2) leaf mining caused by flea beetle larvae (*Phyllotreta nemorum*) and leaf mining flies and (3) leaf removal caused by chewing herbivores such as caterpillars, grasshoppers and slugs (e.g., 80% shot holes, 20% chewing). The herbivores that were spotted on the plants were identified where possible to species level or to genus/family level. In total, the experiment ran for four months, and was ended when the first plants completely filled up the cages. This was done to avoid that with time plant growth would be limited by cage size (a ‘cage saturation’ effect). The aboveground biomass was harvested, dried at 60 °C for 4 days and weighed.

### Chemical analysis

Leaf samples were taken from the Wageningen (NL) and Průhonice (CZ) experimental sites at the time of shoot biomass harvest at the end of the experiment. Due to technical limitations, this was not possible at the site in Germany. Two leaves per plant, the fourth and fifth leaves from the top, were harvested, directly flash-frozen in liquid nitrogen, freeze-dried, and stored at −20 °C until chemical analyses. Samples were pooled per population and herbivory treatment to reduce the amount of samples. This resulted in 48 samples in total (12 populations × 2 herbivore treatments × 2 sites).

For glucosinolate analyses, the freeze-dried leaf samples were ground with a mortar and pestle, 75 mg dry material was weighed in a 2 ml Eppendorf tube and extracted twice with 1 ml of 70% methanol solution and 15 min of ultra-sonification. During the first extraction round, the tube was placed in a hot (90 °C) water bath for 10 min, right after the addition of the methanol to immediately kill myrosinase activity. After the sonification, the tubes were centrifuged at 4500 rpm (2975 rcf) for 10 min. The supernatants were combined and added to a DEAE Sephadex A 25 column (Sigma-Aldrich). After washing the columns with 2 × 1 ml 70% MeOH and 2 × 1 ml MilliQ water and 1 × 1 ml NaOAC buffer (20 mM, pH = 5.5.), 20 μl containing 10 units arylsulfatase (Sigma-Aldrich) was added to the column and left overnight. The desulphated glucosinolates were eluted from the column with 2 × 0.75 ml milliQ water. The combined eluate was freeze dried and redissolved in 1.0 ml MilliQ. The desulphoglucosinolates were separated using high-performance liquid chromatography (DIONEX summit HPLC, DIONEX, Sunnyvale, CA, USA) on a reversed phase C-18 column (Alltima C-18, 150 mm length x 4.6 mm o.d., 3 μm particle size, Alltech, Deerfield, IL, USA) with an acetonitrile–water gradient (2–35% acetonitrile from 0 to 30 min; flow 0.75 ml min^−1^). Detection was performed with a photodiode array detector (PDA) and peaks were integrated at 229 nm. Sinigrin was used as an external standard. Five reference sinigrin samples in the range 50–650 μM were used to generate a calibration curve. We used the correction factors for detection at 229 nm from Buchner (1987) and Brown et al. (2003) to calculate the concentrations of the different types of glucosinolates based on the sinigrin reference curve. Glucosinolates were identified based on retention time, UV spectrum, LC–MS analysis of selected reference samples and commercially bought standards.

### Statistical analysis

Statistical analyses were performed with SPSS 22 (IBM). The effect of experimental site, herbivory treatment (open or closed cages) and region of plant origin on plant growth and total herbivore damage were tested with linear mixed models. Experimental site, herbivory treatment and region of plant origin were added as fixed factors; population was added to the model as random factor nested in region of origin. The x and y coordinates of plant position in the experimental plots were added as covariates (row and column number). Data were transformed where needed to meet the assumptions of normality and homoscedasticity [shoot biomass and no. shoots: square root, no. of flower stalks: ranks, percentage damage: arcsine (August measurement) and ranks (June measurement)]. Significant differences between groups were tested with Tukey post hoc tests. Differences between regions of plant origin in spider mite infections were tested with similar mixed models but for the Czech site only [data were log (June measurement) and arcsine (August measurement) transformed]. Due to interdependency of the data, the effect of experimental site and region of plant origin on the proportion of leaf damage types, i.e. proportion mining, shot holes or chewing damage, were analyzed with MANOVA models on rank transformed data. In these MANOVA models, the random effect population was not included. The effect of experimental site, plant origin and herbivory treatment on the concentrations of the individual glucosinolates (μM/mg dw) was tested with the linear nested mixed models described above. In addition, differences in glucosinolate composition were analyzed with a Principal Coordinate Analysis (PCoA) on the relative abundances of individual glucosinolates (percentage of total glucosinolate concentration) in R 3.3.0 (http://www.R-project.org).

## Results

### Survival

Most plants emerged from the root cuttings, 3–5% did not emerge at the German and Czech experimental sites, 8% at the Dutch site. All plants survived throughout the experiment at the German and Czech sites, 15% died at the Dutch site in novel range during the experiment and mortality was equal among the plant origins. In total, 649 plants of 720 were alive at the end of the experiment.

### Regional adaptation and plant performance

**Fig. 2:**
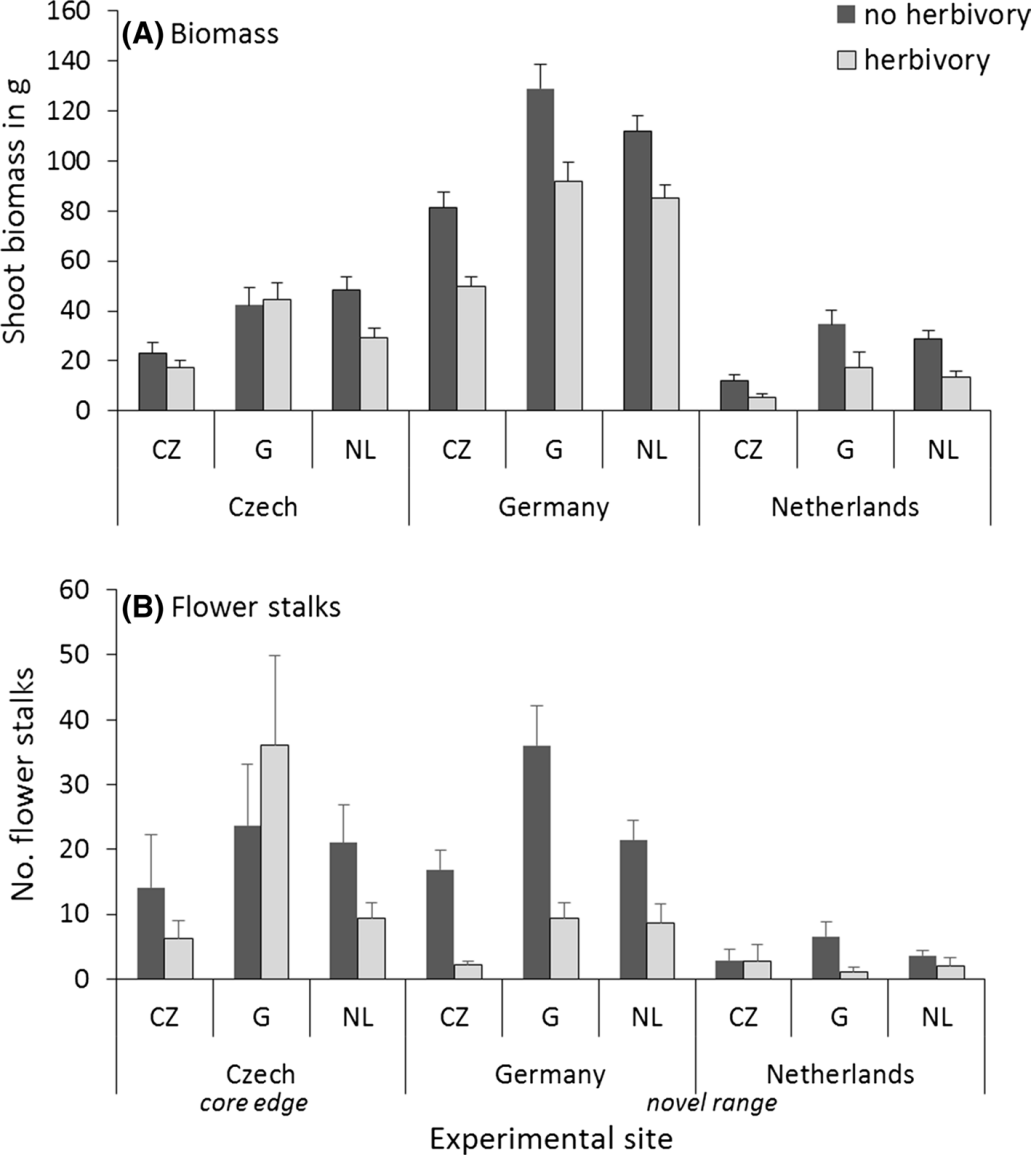
**a** Shoot biomass (*g* ± SE) and **b** number of flower stalks in August (±SE) of *Rorippa austriaca* at the experimental sites in the core range edge (Czech Republic) and the newly colonized range (Germany and the Netherlands). *Bars* indicate means of plants originating from the core edge Czech region (CZ, five populations) and from regions in the novel range in Germany (*G*, two populations) and the Netherlands (*NL*, five populations). *Light gray bars* indicate the means with herbivory; *dark gray* bars indicate means when herbivores were excluded. *N* = 649. For statistics, see Table 2. For data on populations, see Supplementary Fig S1

Experimental site had a significant effect on all measured plant traits (Table 1). Overall, plants were the smallest at the Dutch experimental site and the largest at the German site, which was reflected throughout the growing season in plant height, shoot number, number of flower stalks and final shoot biomass (Table 1; Fig. 2, Fig S1). Region of plant origin (Czech Republic, Netherlands, Germany) also had a significant overall effect on most of the plant traits, except on herbivore damage (Table 1). At all sites, the Czech plants from the core edge were smaller than the plants from the novel range (German and Dutch origins) which were equally big (Table 1; Fig. 2, post hoc Tukey tests *P* < 0.0001 for all G-CZ and NL-CZ comparisons, *P* > 0.52 for G-NL comparisons).

**Table 1:** *F* values of experimental site, region of plant origin and herbivory treatment effects on plant performance traits and damage in June and August (mixed models)

Factor	*df*	Shoot biomass	Shoot length		No. shoots		No. flower stalks		Damage	
			June	August	June	August	June	August	June	August
Site	2	342.64***	120.22***	41.55***	269.45***	263.01***	72.59***	57.86***	186.17***	7.89**
Plant origin	2	46.31***	9.86**	62.48***	0.66	4.54*	6.12*	6.48*	2.02	1.95
Herbivory	1	57.89***	44.20***	56.21***	17.39**	17.71***	19.36**	48.04***	100.04***	427.70***
Site × origin	4	0.31	0.89	1.34	0.51	0.44	2.95*	1.44	1.28	1.08
Origin × herbivory	2	0.37	3.18	0.82	0.31	0.45	0.29	0.29	0.62	1.86
Site × herbivory	2	4.01*	6.90**	4.06*	2.65	1.92	17.35***	10.57***	62.84***	10.66***
Origin × site × herbiv.	4	1.59	0.64	1.48	0.78	0.27	0.16	1.72	0.10	2.27
*X*-coordinate	1	5.89*	0.29	21.26***	0.2	3.69	5.59*	1.32	2.88	3.37*
*Y*-coordinate	1	25.24***	16.42***	16.94***	0.45	0.02	2.94	0.57	0.98	0.25

Bold values indicate signficant role by 3 factors site, origin and herbivory

Models included plant population as a random factor nested in origin and its interaction with site and treatment (not shown). x and y coordinates of plant position in the experimental plots were added as covariates. *N* = 649

*** *P* < 0.0001, ** *P* < 0.005, * *P* < 0.05

A significant interaction between environment and genotype can be an indication for adaptation to local environments. Here, the interaction between experimental site x region of plant origin was not significant for any of the measured traits (Table 1), except for number of flower stalks in June. This general lack of significant site x plant origin interaction indicates no local advantage and thus no regional adaptation of the *R. austriaca* populations. As for flower production in June, no flowers were produced at the Dutch site then. At German site at that time, number of flower stalks did not differ among plants from the different regions (post hoc Tukey, *P* > 0.05). At the Czech site in the core edge, the Czech plants produced less flower stalks than the Dutch plants in June (post hoc Tukey *P* = 0.007). In August, the Czech plants produced less flower stalks than the Dutch and German plants at the Czech and German sites (post hoc Tukey tests *P* < 0.01) (Fig. 2; Table 1). Although the interaction between experimental site × region of plant origin was significant for this trait in June, the results do not indicate a local *vs.* foreign advantage, but rather a local disadvantage.

### Herbivore impact on plant performance

Herbivory significantly reduced shoot biomass on average by 26% at the Czech site, 32% at the German site and 54% at the Dutch site (Table 1; Fig. 2, comparison of shoot biomass without and with herbivory, post hoc tests for effect of herbivory treatment within site, *P* = 0.008 for Czech site, *P* < 0.0001 for the Dutch and German sites). The biomass of the German populations was not affected by herbivory at the Czech site (Fig. 2).

The experimental site × herbivory treatment interaction was significant for most plant traits, see Table 1. This was due to the lower impact of herbivory on plant performance at the core edge Czech site compared to the other sites. However, there was no significant interaction between region of plant origin and herbivory treatment for any measured trait (Table 1). There was no significant three-way interaction between experimental site, plant origin and herbivory treatment (Table 1). The pattern that the core edge plants were smaller than the plants from the novel range remained the same with and without herbivory at all sites.

### Herbivore damage

Overall herbivore leaf damage in the herbivory treatment was the lowest at the core edge Czech site and highest at the Dutch site in the novel range (post hoc Tukey test *P* < 0.05, Tables 1 and 2), where damage on the leaves was around 50% in June.

**Table 2:** Average percentage herbivore damage (SE) on *Rorippa austriaca* plants from three regions of origin—core edge Czech Republic (CZ), and the newly colonized areas Germany (G) and the Netherlands (NL)—at the experimental sites in each region

Site	Plant origin	*N*	June	August
Czech	CZ	42	0.5 (0.2)	13.3 (2.6)
	G	20	0.3 (0.1)	14.1 (4.0)
	NL	50	0.2 (0.1)	12.5 (2.3)
Germany	CZ	48	13.5 (1.6)	19.0 (2.0)
	G	20	11.4 (2.7)	15.5 (2.3)
	NL	48	10.1 (1.1)	12.5 (0.7)
The Netherlands	CZ	35	67.6^a^ (5.2)	31.2^a^ (4.4)
	G	14	57.8^ab^ (7.8)	15.2^b^ (2.9)
	NL	38	51.5^b^ (4.4)	23.9^ab^ (3.1)

Data shown of the herbivory treatment only (open cages). Herbivore damage in the closed cages was on average 3% (±1%) in August for each experimental site and plant origin

Different letters indicate significant differences between plant origins at a site (post hoc Tukey tests *P* < 0.05)

At the Czech and the German sites, the amount of damage was the same among the regions of plant origin (Table 2). At the Dutch site in the novel range, the Czech plants from the core were eaten more compared to plants from the novel range of Germany (in August) or the Netherlands (in June) (Table 2, post hoc Tukey test *P* < 0.05). Plant biomass was negatively correlated with the amount of herbivore damage (plants in herbivory treatment: *R*_s_ = −0.15, *N* = 315, *P* = 0.008).

### Herbivore composition and type of damage

**Fig. 3:**
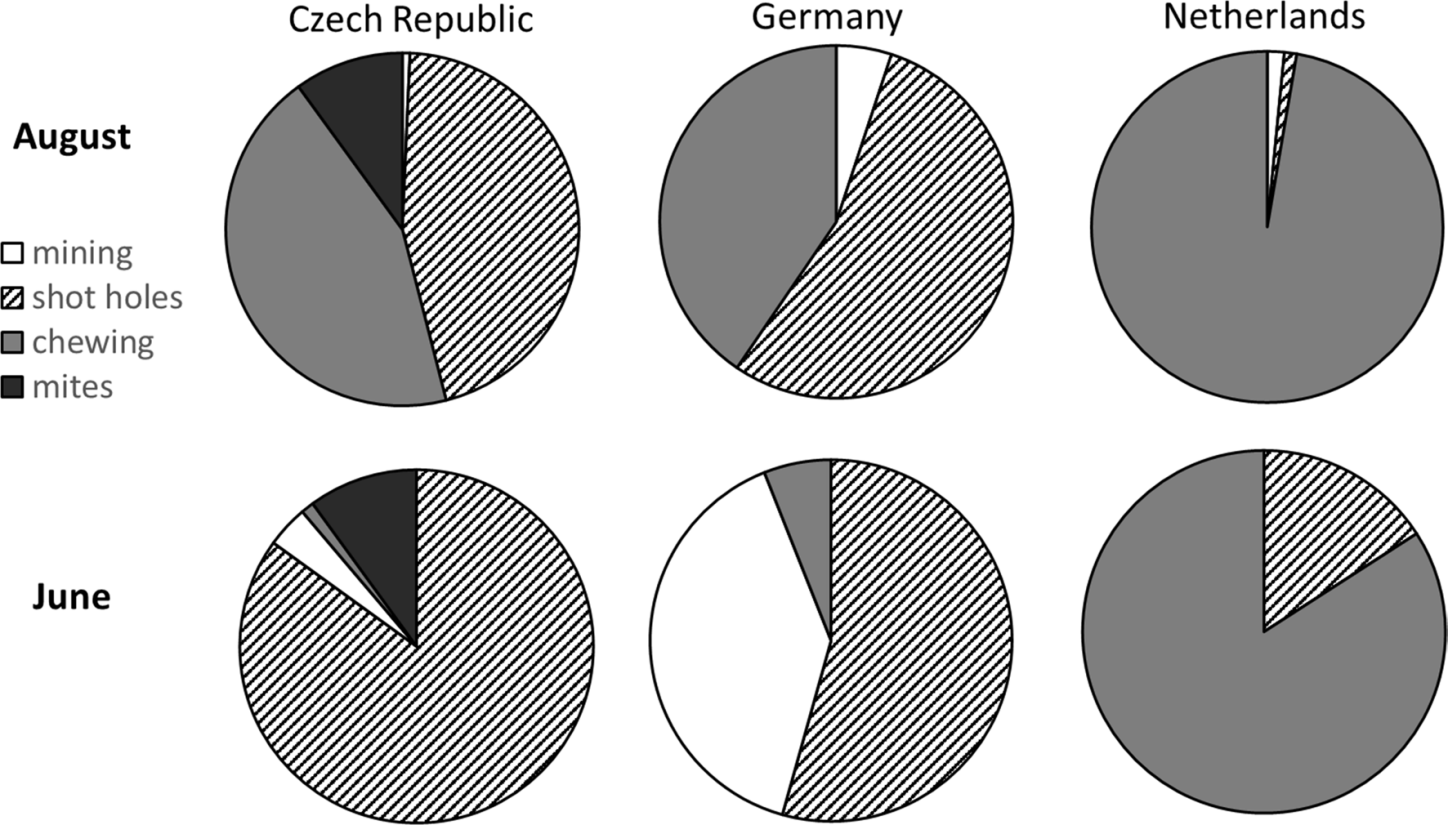
Composition of types of leaf damage at the experimental transplant sites, based on mean percentages of the total damage (*N* = 109–115 per site). Shot holes: damage by adult flea beetles (Chrysomelidae), mining: damage by flea beetle larvae, chewing: damage by chewing herbivores such as slugs, snails and caterpillars. Differences between sites were significant (*P* < 0.0001, MANOVA on ranks, Table S2)

The composition of the type of herbivore leaf damage was different between the experimental sites. At the Dutch site in the novel range, there was mostly chewing damage, while at the Czech and German sites damage by adults and larvae of chrysomelid beetles was most common (Fig. 3; Table S2). Herbivore species observed on the plants were similar between the Czech and the German sites, except for infections of spider mites which only occurred at the Czech site and which was equal among plant origins (*P* > 0.11, mean 10%) (Table 3). The Dutch site was dominated by slugs.

**Table 3:** Herbivores recorded on *Rorippa austriaca* at the three experimental sites

Herbivores	Novel range		Core edge
	Tübingen, Germany (G)	Wageningen, Netherlands (NL)	Průhonice, Czech Republic (CZ)
*Phyllotreta nemorum* (Coleoptera; Chrysomelidae)	XX		X
Chrysomelidae other	X	X	X
*Eurydema oleraceum* (Hemiptera; Pentatomidae)	X		X
*Lygus* sp. (Hemiptera; Miridae)	X		X
*Pieris rapae* (Lepidoptera; Pieridae)	X		X
*Pieris brassicaea* (Lepidoptera; Pieridae)	X		X
*Plutella xylostella* (Lepidoptera; Plutellidae)	X		X
*Autographa gamma* (Lepidoptera; Noctuidae)	X	X	X
*Brevycoryne brassiceae* (Hemiptera; Aphididae)	X	X	X
Aphids other	X	X	X
Thrips (Thrysanoptera)	X	X	X
Spider mites (Arachnidae; Tetranychidae)			XX
Slugs	X	XX	
Snails	X	X	X
Grasshoppers (Orthoptera; Acrididae)	X	X	X
Leafhoppers (Hemiptera; Cicadellidae)	X	X	X

X indicates present; XX indicates high abundance (Macel and Dostalek, personal observation)

### Chemical defenses

**Fig. 4:**
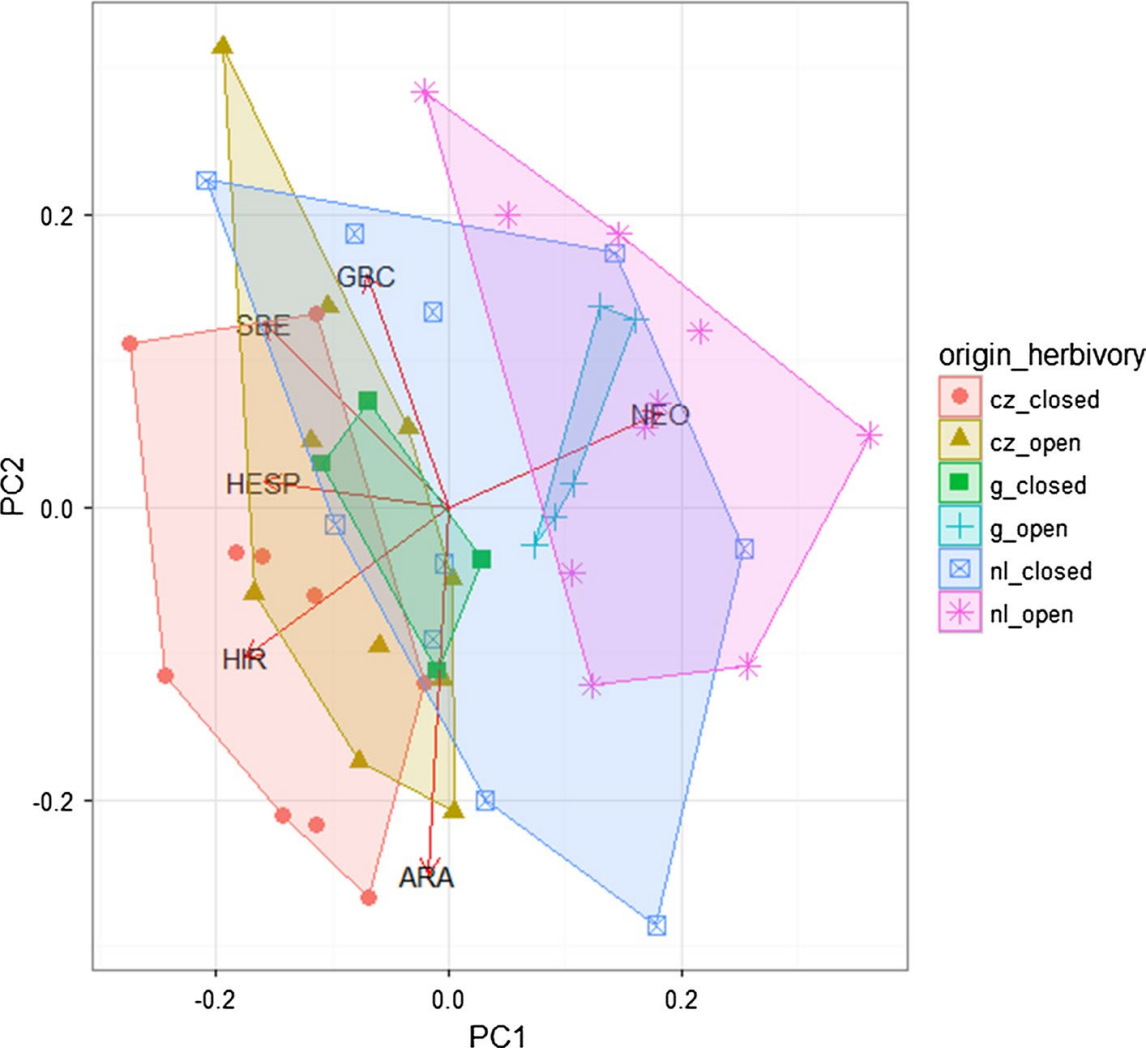
PCA plot of glucosinolate profiles (based on relative abundance) of *Rorippa austriaca* populations from the native (cz) and the new range (*g*, nl). Plants without herbivory (*closed cages*) and with herbivory (*open cages*). Different colors indicate different regions of plant origin × herbivory treatment; experimental site (NL and CZ) is not indicated. Samples from plants of one population × herbivory treatment per site were pooled. *n* = 49. PC1 explains 51% of the variance, PC2 28%. *NEO* neoglucobrassicin, *ARA* glucoarabin, *HIR* glucohirsutin, *HESP* glucohesperin, *SBE* glucosiberin, *GBC* glucobrassicin

The analyses of the chemical defenses, the glucosinolates, showed small differences between the plant origins, particularly among plants in the herbivory treatment (Fig. 4, Table S3). Dutch populations from the novel range had overall higher amounts of the indole glucosinolate neoglucobrassicin (NEO) and lower levels of glucohirsutin (HIR) compared to the core edge Czech plants (Fig. 4, Table S3, Fig S2). Neoglucobrassicin was induced by the herbivory treatment, especially in the plants from the novel range (Fig. 4; Table S3 significant herbivory treatment × plant origin interaction, Fig. S2). Total glucosinolate concentrations did not differ between the plant origins. Site had a significant effect on the concentrations of most glucosinolates. Concentrations were higher at the Dutch site (Table S3; Fig S2).

Using average estimates of traits (glucosinolates, biomass) for each population (12 populations in total), there were no significant correlations between total glucosinolate concentrations and plant biomass for any of the experimental site x treatment combinations. Within the herbivory treatment, at the core edge Czech site damage was positively correlated with total glucosinolate levels (*R*_s_ = 0.62, *N* = 12, *P* = 0.031) and with glucohirsutin levels (*R*_s_ = 0.70, N = 12, *P* = 0.01). In contrast, at the Dutch site neoglucobrassicin was negatively correlated with damage (*R*_s_ = −0.61, *N* = 12, *P* = 0.03) (Fig S3 and S4).

## Discussion

Here, we tested whether a range expanding plant has rapidly adapted to the regional conditions in its novel range and if so, whether such adaptation was driven by interactions with herbivores. Our results indicate that there is no regional adaptation to abiotic conditions such as climate, although we did not test adaptation to local soil types and only examined a relatively short window of the entire life span of this perennial plant. Interestingly, herbivore damage was not lowest but highest at the sites in the new range, and our data suggest that plants from the novel range have evolved increased herbivore resistance and increased vigor. Plants originating from the newly colonized regions in the Netherlands and Germany were larger than the core edge Czech plants at all sites. Most plants were negatively affected by herbivory but also under herbivory plants from the novel range grew larger than the core edge plants at all sites. This indicates genetic differentiation in size between plants from the novel range and core plants which is independent of environmental factors. There was also a plastic response to the different environmental conditions at the three sites, as plants grew overall biggest at the German site and were smallest at the most northern site in the Netherlands.

Increased size or vigor of exotic genotypes compared to genotypes of the native range has often been found in introduced invasive species (e.g., Willis et al. 1999; Torchin et al. 2001; Jakobs et al. 2004; Abela-Hofbauerova and Munzbergova 2011; but see Parker et al. 2006). One of the popular hypotheses that offers an evolutionary explanation for this is the Evolution of Increased Competitive Ability (EICA) hypothesis (Blossey and Nötzold 1995). In the introduced range plants that invest less in defense and more into growth can be favored through enemy release, which enables a shift in allocation from defense to growth. In our experiment, however, herbivory was more and not less severe at the two sites in the novel range compared to the core edge site. Release from enemies is thus not a likely explanation for the observed increased vigor of the populations of the novel range although we only tested one core site and one growing season. The increased vigor of *R. austriaca* from the novel range was consistent with the results of other experiments using the same populations (Dostalek et al. 2016; J. Cross and M. Huberty, unpublished data). This increased vigor is also consistent with other findings from invasive *R. austriaca*: exotic North American *R. austriaca* were also larger compared to native populations but herbivore resistance (to slugs) was the same between the European and North American origins (Buschmann et al. 2005). These results suggest that other mechanisms are responsible for the increased plant growth of *R. austriaca* in its introduced or expanding range.

For invasive species, alternative explanations to enemy release and the EICA hypothesis for the increased vigor of exotic genotypes have been formulated (Colautti et al. 2004). One possibility could be admixture whereby genotypes that were previously isolated in the native range hybridize in the new range which can lead to increased vigor of the offspring (hybrid vigor) (Ellstrand and Schierenbeck 2006; Verhoeven et al. 2011). In addition, hybridization and genetic introgression in the *Rorippa* genus is common (Bleeker and Hurka 2001). We excluded hybrids from our study but we cannot be completely sure that there was no genetic introgression in any of the *R. austriaca* genotypes we tested. Another possibility could be that there is a trade-off between growth and another trait (not herbivore resistance). For example, in an additional experiment, the Dutch populations of the novel range showed no allelopathic effects while the core edge Czech populations had a negative allelopathic effect on a co-occurring grass species (Dostalek et al. 2016). Possibly, there is a trade-off between allelopathy and growth, and loss/lack of allelopathy is accompanied by increased growth in the populations of the novel range.

Range expanding plants have been shown to lose a part of their specialist enemies from the core range (Lakeman-Fraser and Ewers 2013), but similar to introduced invasive species they are also likely to encounter new generalist herbivores which can provide biotic resistance of the native communities towards invading exotic species (Parker et al. 2006; Brown and Vellend 2014; Alofs and Jackson 2015). Strikingly, herbivores had a higher impact on *R. austriaca* performance in the novel range compared to the core edge in our experiment. Our most Northern site in the novel range (Wageningen, The Netherlands) with most herbivore damage (up to 67%) was dominated by generalist slug herbivory. Slug herbivory is likely to be important for determining plant abundance in Northern European regions with high rainfall (Bruelheide and Scheidel 1999). The South German site in the novel range had similar insect herbivore communities to core edge Czech site, but there was more damage earlier on in the season in Germany. The presence of Brassicaceae specialist insects was higher at these southern sites compared to the more northern Dutch site. However, most, if not all, insect species listed at the German and Czech sites in Table 2 are also currently present in the Netherlands, although not on *R. austriaca* at our Dutch experimental site (www.soortenbank.nl ETI bioinformatics). Thus, even though our results could be due to a local site effect, it also shows that damage from generalist herbivores can be severe in the northern new range. Similar patterns of more herbivory in the extended range were found for range expanding algae (Nylund et al. 2012). Experiments with replicated sites at each latitude would indicate whether the herbivory pattern that we found here is a general latitudinal pattern.

Differentiation in herbivore resistance between native and exotic populations of introduced invasive plants is common (Doorduin and Vrieling 2011; Felker-Quinn et al. 2013), but evolution of resistance/defenses is not well documented for range expanding plants. In range expanding algae, resistance towards generalist herbivores was lower in core populations to populations in the extended range (Nylund et al. 2012). In contrast, resistance against a generalist caterpillar was higher in core populations of the range expanding plant *Bunias orientalis* (Fortuna et al. 2014). Interestingly, our results suggested that core edge Czech *R. austriaca* were less resistant to herbivory than the German and Dutch populations of the novel range at the slug dominated north-western site. Differentiation in chemical defenses could play a role in this resistance. Slugs are known to be sensitive to glucosinolates (Lankau 2007; Falk et al. 2014; Moshgani et al. 2014). Specialist herbivores, on the other hand, can use glucosinolates as feeding and oviposition cues (Renwick et al. 1992). Overall levels of glucosinolates were similar between *R. austriaca* from the core edge and the novel range, but glucohirsutin was higher in the core populations and neoglucobrassicin was higher in the populations of the novel range. Correlations between damage and these two compounds were opposite between the core Czech site and the Dutch site in the novel range. Therefore, these subtle differences in glucosinolate profiles between the *R. austriaca* origins could indicate shifts in selection by herbivores for individual glucosinolates (Gols et al. 2008; Züst et al. 2012). There was also a stronger induction of neoglucobrassicin in the genotypes from the novel range compared to the core genotypes. Upon attack plants can induce the levels of chemical defenses (Karban and Baldwin 1997). Higher inducibility of defenses of exotic populations compared to native populations has received little attention thus far, but this could be an adaptation to lower or less predictable enemy attack in the introduced or extended range (Cipollini et al. 2005; Cipollini and Lieurance 2012; Gu et al. 2014; Fortuna et al. 2014).

Species that are expanding their range to higher latitudes can include potential invaders that are less affected by herbivores compared to native species (Engelkes et al. 2008). Here, we showed that these range expanders can also have increased vigor in the extended range compared to core populations, a trait often found in invasive species. This increased growth was accompanied by subtle shifts in defenses and higher inducibility of a particular defense compound. Mechanisms behind this differentiation of the novel populations remain to be elucidated. *R. austriaca* can be abundant in riverine areas in its novel range (Bleeker 2003) and is considered a weed in agriculture in its core distribution. From a biological conservation perspective, it is desirable that species are able to shift their ranges in response to climate change, because it is one of the ways for species to possibly avoid extinction (Berg et al. 2010). However, these range expanding species may include potential invaders or could evolve invasive traits in their novel range. Theories that have been applied to explain invasive success of exotic species from other continents could perhaps (partly) also be applied to the successful spread and abundance of these range expanders, a process about which we still know very little (Moran and Alexander 2014).

## Electronic supplementary material

Below is the link to the electronic supplementary material.

Supplementary material 1 (DOCX 241 kb)
