# Supplementary material for: Evolutionary responses to climate change in a range expanding plant
Source: Oecologia. 2017 Apr 13;184(2):543–54. doi: 10.1007/s00442-017-3864-x (PMC5487849; doi:10.1007/s00442-017-3864-x)

**Electronic Supplemental Material (ESM)**

Table S1. Origin, code, coordinates and habitat of the *Rorippa austriaca* populations used in the experiments

| Range | Region | Population | Code | Latitude | Longitude | Habitat |
| --- | --- | --- | --- | --- | --- | --- |
| Core edge | Czech Republic | Moravské Budějovice | CMB | 49˚02'47"N | 15˚48'01"E | Ditch |
|  | Czech Republic | Modřany - Na Cikorce | CMC | 50˚00'35"N | 14˚24'53"E | grassland |
|  | Czech Republic | Modřany - U jezu | CMJ | 50˚00'17"N | 14˚24'07"E | river bank |
|  | Czech Republic | České Budějovice | CCB | 48˚58'41"N | 14˚26'41"E | road side |
|  | Czech Republic | Hluboká nad Vltavou | CHV | 49˚03'24"N | 14˚26'44"E | grassland |
| Novel | Germany | Rastatt | GRS | 48˚52’15"N | 8˚12’32"E | grassland |
|  | Germany | Plittersdorf | GPL | 48˚53’40"N | 8˚09’08"E | grassland |
|  | Netherlands | Rotterdam | NRD | 51˚57'58" N | 4˚27'15"E | road side |
|  | Netherlands | Meinerswijk | NMW | 51˚58’56"N | 5˚52.08"E | river bank |
|  | Netherlands | Millingerwaard | NML | 51˚51'52"N | 5˚59'15"E | river bank |
|  | Netherlands | Maassluis | NMS | 51˚55'49"N | 4˚13'37"E | river bank |
|  | Netherlands | Wageningen | NWG | 51˚57'15"N | 5˚39'24"E | river bank |

Table S2. MANOVA results for the effects of experimental site (Czech Republic, Germany and Netherlands) and region of plant origin on the type of leaf damage on *Rorippa austriaca* (estimated as percentage of all damage). Table entrees are *F* values. *N* = 109-115 per site. Data were rank transformed.

| **Factor** | ***Df*** | **Shot holes June** | **Shot holes August** | **Chewing June** | **Chewing August** | **Mining June** | **Mining August** |
| --- | --- | --- | --- | --- | --- | --- | --- |
| Site | 1 | 77.50*** | 72.08*** | 214.85*** | 170.79*** | 115.46*** | 15.49*** |
| Origin | 2 | 0.98 | 0.68 | 0.35 | 3.58 | 0.10 | 0.57 |
| Site x Origin | 4 | 0.75 | 0.52 | 0.75 | 0.29 | 0.25 | 2.10 |

*** P < 0.0001

Table S3. Mixed-model results for the effects of experimental site (Czech Republic and Germany), herbivory treatment and region of plant origin on glucosinolate concentrations (μM/mg dry weight). Models included population as random factor nested in origin. Table entrees are *F* values. *N* = 48 (plants per population x herbivory treatment were pooled).

| **Factor** | ***df*** | **Total** | **HESP** | **SBE** | **GBC**^+^ | **HIR** | **ARA** | **NEO** |
| --- | --- | --- | --- | --- | --- | --- | --- | --- |
| Site | 1 | 4.98* | 5.82* | 31.19*** | 257.51*** | 3.38 | 9.35** | 0.02 |
| Herbivory | 1 | 3.63 | 0.11 | 0.05 | 1.61 | 0.58 | 1.79 | 79.99*** |
| Origin | 2 | 0.55 | 1.56 | 0.68 | 1.12 | 4.36* | 2.59 | 18.12** |
| site x herbivory | 1 | 1.66 | 0.15 | 0.06 | 1.41 | 0.21 | 2.74 | 19.43** |
| site x origin | 2 | 1.39 | 6.45** | 1.30 | 0.88 | 1.82 | 3.44* | 0.94 |
| herbivory x origin | 2 | 0.74 | 1.01 | 0.59 | 0.79 | 0.46 | 0.15 | 5.76* |
| site x herbivory x origin | 2 | 0.09 | 0.84 | 0.54 | 3.95* | 0.55 | 0.21 | 2.54 |

HESP = glucohesperin, SBE = glucosiberin, GBC = glucobrassicin, HIR = glucohirsutin, ARA = glucoarabin, NEO = neoglucobrassicin

^+^ log transformed data

* P < 0.05, ** P < 0.005, *** P < 0.0001

Fig. S1. Biomass per population per treatment per experimental site


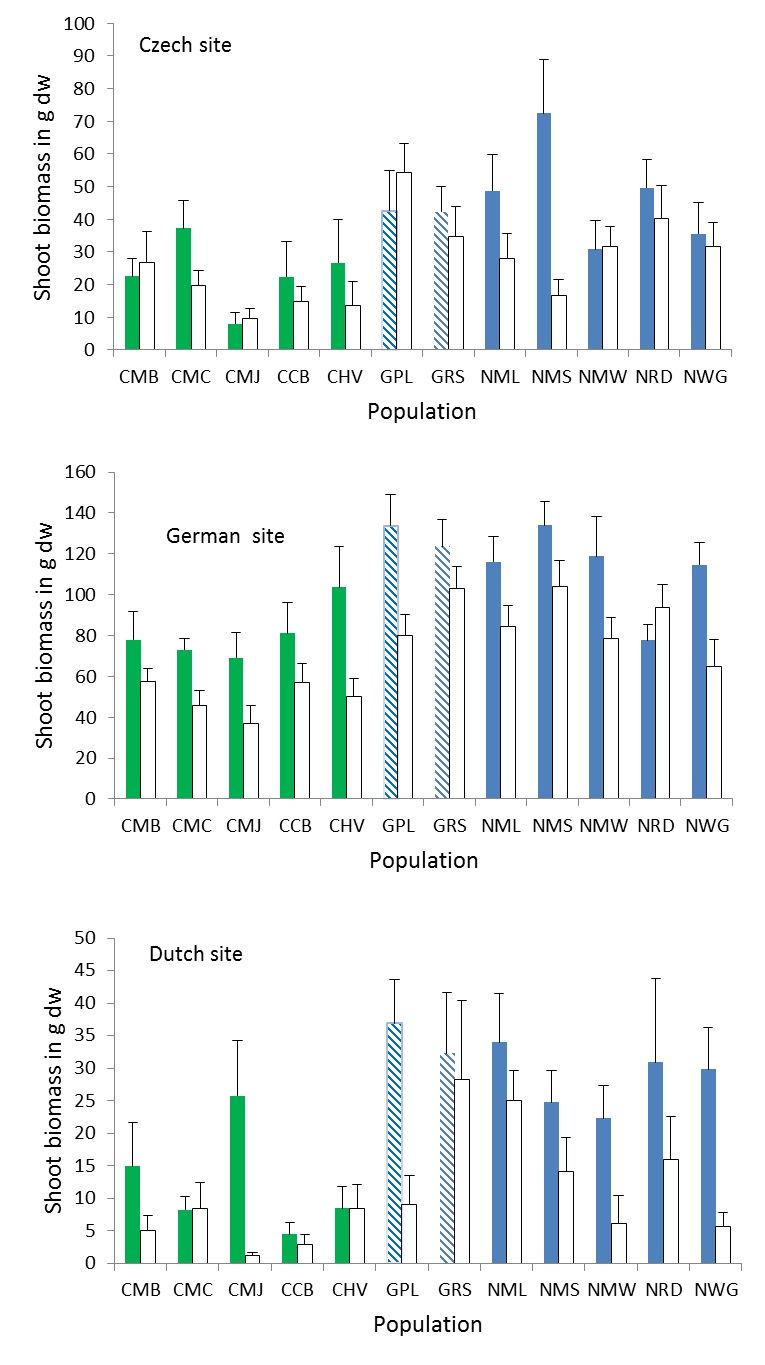


Fig. S2 glucosinolate profiles of plants of the three regions in two experimental sites, Pruhonice (Czech Republic) and Wageningen (the Netherlands). concentrations per glucosinolate in µM/mg dry weight. HESP = glucohesperin, SBE = glucosiberin, GBC = glucobrassicin, HIR = glucohirsutin, ARA = glucoarabin, NEO = neoglucobrassicin


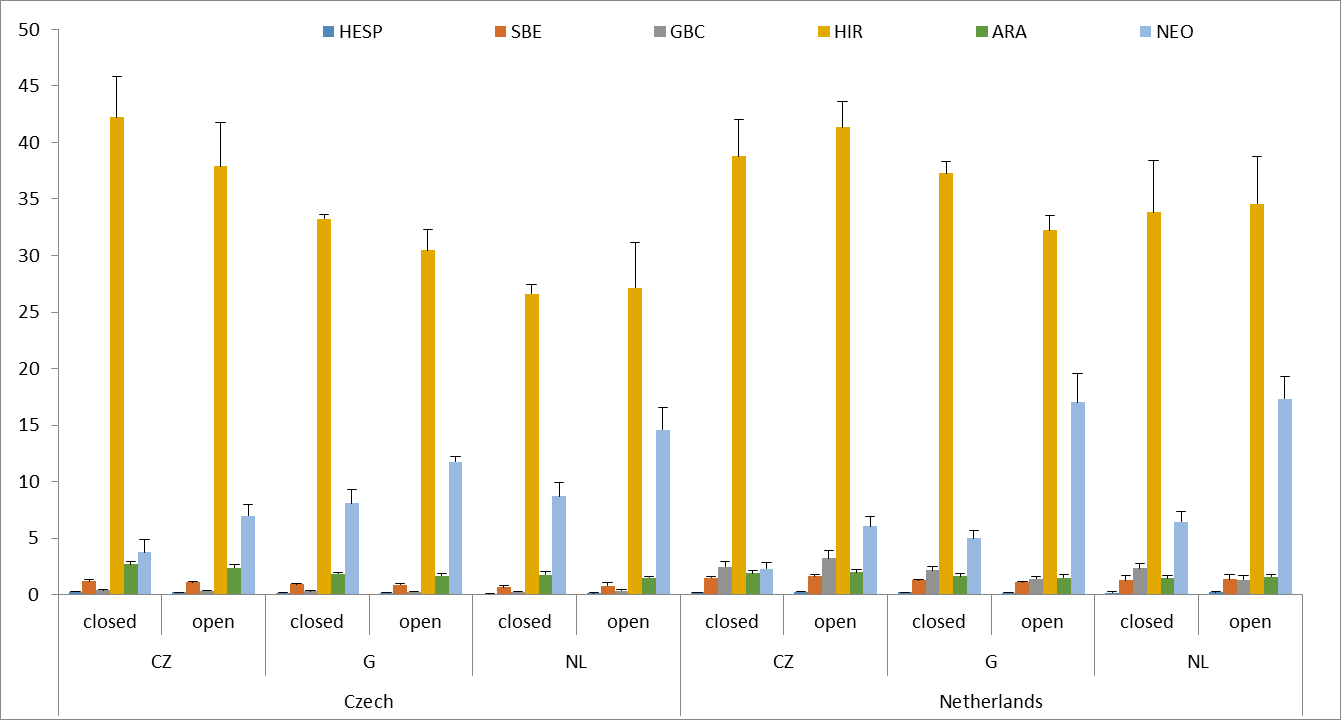


Fig. S3. Correlation between percentage damage and total glucosinolates of *Rorippa austriaca* at Pruhonice, Czech Republic (CZ) and Wageningen, the Netherlands (NL) experimental sites. Herbivory treatment only.

Fig. S4. Correlation between percentage damage and neoglucobrassicin (NEO) and between damage and glucohirsutin (HIR) of *Rorippa austriaca* at Pruhonice, Czech Republic (CZ) and Wageningen, the Netherlands (NL) experimental sites. Herbivory treatment only.


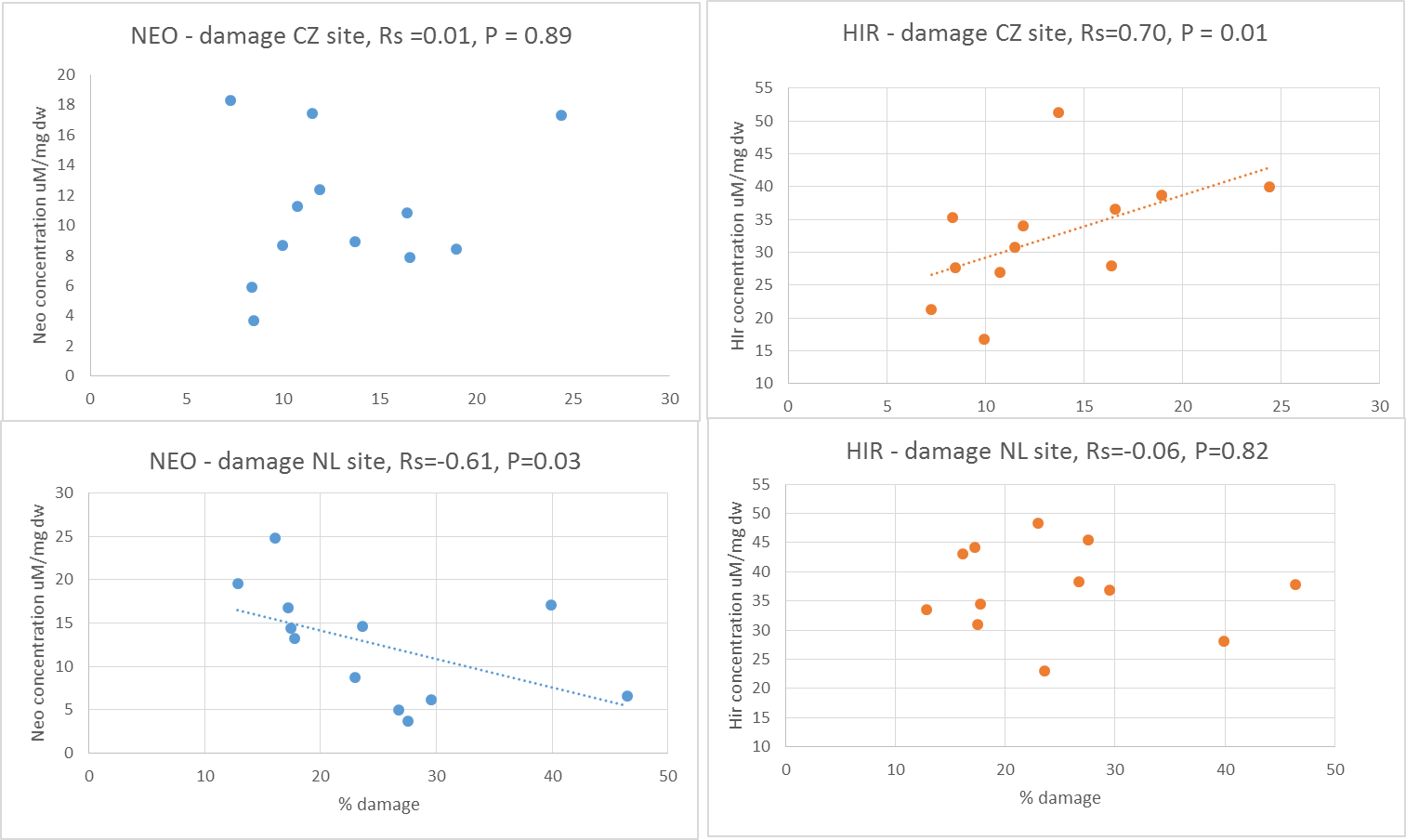

Supplement: Supplementary file 1 — Supplementary material 1 (DOCX 241 kb) [file 442_2017_3864_MOESM1_ESM.docx]
